# Exploring the Potential of Electrical Impedance Tomography for Tissue Engineering Applications

**DOI:** 10.3390/ma11060930

**Published:** 2018-05-31

**Authors:** Hancong Wu, Wenli Zhou, Yunjie Yang, Jiabin Jia, Pierre Bagnaninchi

**Affiliations:** 1 Agile Tomography Group, School of Engineering, The University of Edinburgh, Edinburgh EH9 3JL, UK;hason.wu@ed.ac.uk (H.W.); y.yang@ed.ac.uk (Y.Y.); jiabin.jia@ed.ac.uk (J.J.); 2 Department of Medical Oncology, Changzheng Hospital, Navy Medical University, Shanghai 200070, China; Zhouwenli@smmu.edu.cn; 3 MRC Centre for Regenerative Medicine, The University of Edinburgh, Edinburgh EH16 4UU, UK

**Keywords:** electrical impedance tomography, tissue engineering, cell viability, scaffolds, hydrogels

## Abstract

In tissue engineering, cells are generally cultured in biomaterials to generate three-dimensional artificial tissues to repair or replace damaged parts and re-establish normal functions of the body. Characterizing cell growth and viability in these bioscaffolds is challenging, and is currently achieved by destructive end-point biological assays. In this study, we explore the potential to use electrical impedance tomography (EIT) as a label-free and non-destructive technology to assess cell growth and viability. The key challenge in the tissue engineering application is to detect the small change of conductivity associated with sparse cell distributions in regards to the size of the hosting scaffold, i.e., low volume fraction, until they assemble into a larger tissue-like structure. We show proof-of-principle data, measure cells within both a hydrogel and a microporous scaffold with an ad-hoc EIT equipment, and introduce the frequency difference technique to improve the reconstruction.

## 1. Introduction

Tissue engineering aims to generate artificial tissues and organs to repair or replace damaged tissues in body [1]. It generally uses a combination of cells and biomaterials to engineer samples several orders of magnitude higher than a single cell volume. Currently, tissue engineering makes use of a variety of biomaterials varying from polymers, hydrogels to decellularised animal tissues [2]. Cell viability and proliferation are often difficult to be assessed in large samples with height and diameter at the millimeter scale, and composed of non-transparent biomaterials. Hence, techniques to investigate engineered tissues are at the cross-road between materials characterization, medical imaging and optical microscopy.

Scanning electron microscopy (SEM), transmission electron microscopy (TEM), and micro computed tomography (micro CT) are popular techniques in the field of tissue engineering, and together with confocal and multiphoton microscopy, they have greatly helped us to improve our understanding of tissue formation and cell–material interactions. However, these techniques require fixing and cutting the tissues to perform histology staining, and the samples cannot be used in further studies. MTT and MTS cell proliferation assays are alternative techniques to evaluate the cell viability in the biomaterials. However, these assays are time-consuming and require dyeing [3], so that they cannot perform a real-time and label-free assessment of cell viability in large samples, which will be valuable to control the quality of the artificial tissue before implanting it in the body, without having to use fluorescents or radioactive labels.

It was demonstrated in the early 1900s that cell viability could be inferred by measuring the electrical parameter of cells [4]. The double lipid bilayer membrane acts as an insulating interface, delimiting two conductive media—the cytoplasm and the outer media. Hence, when the membrane is compromised upon cell death, a radical change in cell conductivity occurs. The passive electrical properties of biological cells are frequency dependent due to the interface polarization [4]. To adopt these electrical properties to estimate the cell activities, the main technique is to generate an AC electric field in the cultivation area and to measure the passive electrical response of the cells, which is called dielectric spectroscopy (DS). It is a viable technique for cell suspension and tissue engineering [5,6,7,8], and is therefore a good strategy to monitor cell growth and differentiation state in a non-invasive and label-free way when culturing and differentiating cells in three-dimensional (3D) structures [9]. This was exemplified in the study by Canali et al. [10] to detect the presence of HepG2 cells that were homogeneously distributed in the hydrogel scaffolds. It was also developed as a tool to measure the GFP-MCF-7 cell densities, proliferation and viability in the hydrogel scaffolds using aligned pairs of microelectrodes [11]. Based on the analytical calculation models, the epithelial differentiation processes of Madin–Darby canine kidney (MDCK) cells cultured in 3D printed Poly (DL-lactide-co-glycolide) acid (PLGA) scaffolds filled with collagen gels were monitored by Daoud et al. [12]. Currently, the main concern of DS is that a pair of electrodes only provide a lumped impedance value for the entire hydrogel sample, so the spatial resolution is necessary to be improved [10].

In a medical imaging context, dielectric properties of tissues can be retrieved with spatial resolution with electrical impedance tomography (EIT). Sharing the same theory of the DS, the EIT system allows the users to address multiple measurements from the electrode arrays through a multiplexer for switching the current source and a data acquisition unit [13]. The first EIT system for medical research, the Sheffield Mk1, was developed in 1987 by Brown and Seagar [14]. The system used 16 electrodes, and measurements were performed to monitor the cardiac gating at a fixed frequency of 51 kHz [15]. The second generation of EIT measurement systems was developed during the next two decades. They allowed multi-frequency measurements with the aim to identify biological tissues. Two systems, the UCLH Mark 1b and the KHU Mark 1, were sequentially developed by Yerworth et al. [16] and Oh et al. [17], which achieved the multi-frequency time-difference imaging and frequency-difference imaging of the local small conductivity change in human lungs due to respiration [18].

The EIT was first developed for the clinical applications, such as thorax imaging [19], brain function monitoring [20] and breast cancer screening [21,22], but the development of microtechnologies has recently allowed the application of EIT at the cellular scale. Linderholm et al. [23] developed the first EIT sensor for in vitro experiments, in which 16 parallel planar electrodes were deposited on the substrate of the sensor to monitor the migration of adhesive human epithelial stem cells. Single cell measurements were then carried out to evaluate the cell size [24] and membrane integrity [25]. In this decade, densely packed tumour spheroids have successfully been imaged in proof-of-principle experiments [26] and for the purpose of chemical insult monitoring [27]. It has also been reported that the yeast cell sedimentation in a microchannel can be visualized through the EIT imaging [28].

Previous studies are mainly focused on the time-difference imaging of cell aggregates that have a relatively high concentration in the local area. In this paper, we explored the potential to use the EIT for tissue engineering applications, for which the key challenge was to monitor a sparse cell distribution in regards to the size of the hosting scaffold, i.e., low volume fraction of cells within the scaffold, until they assembled into a larger tissue-like structure. We showed proof-of-principle data, and measured cell distributions within both a hydrogel and a microporous scaffold with a customized EIT equipment. The frequency-difference imaging method and the isotropic total variation (TV) and l1 joint regularization algorithm were applied for the first time in cellular imaging to eliminate the measurement errors, so that the small change of conductivity due to the presence of cells was observed more accurately. Methods to increase the image quality and the potential applications of our proposed method were discussed.

## 2. Materials and Methods 

### 2.1. Principle of EIT

The purpose of EIT measurements is to find the conductivity distribution in a closed domain based on the boundary potential measurements corresponding to the injected current. Its mathematical theory can be summarized in two parts: the forward problem and the inverse problem. 

**Figure 1:**
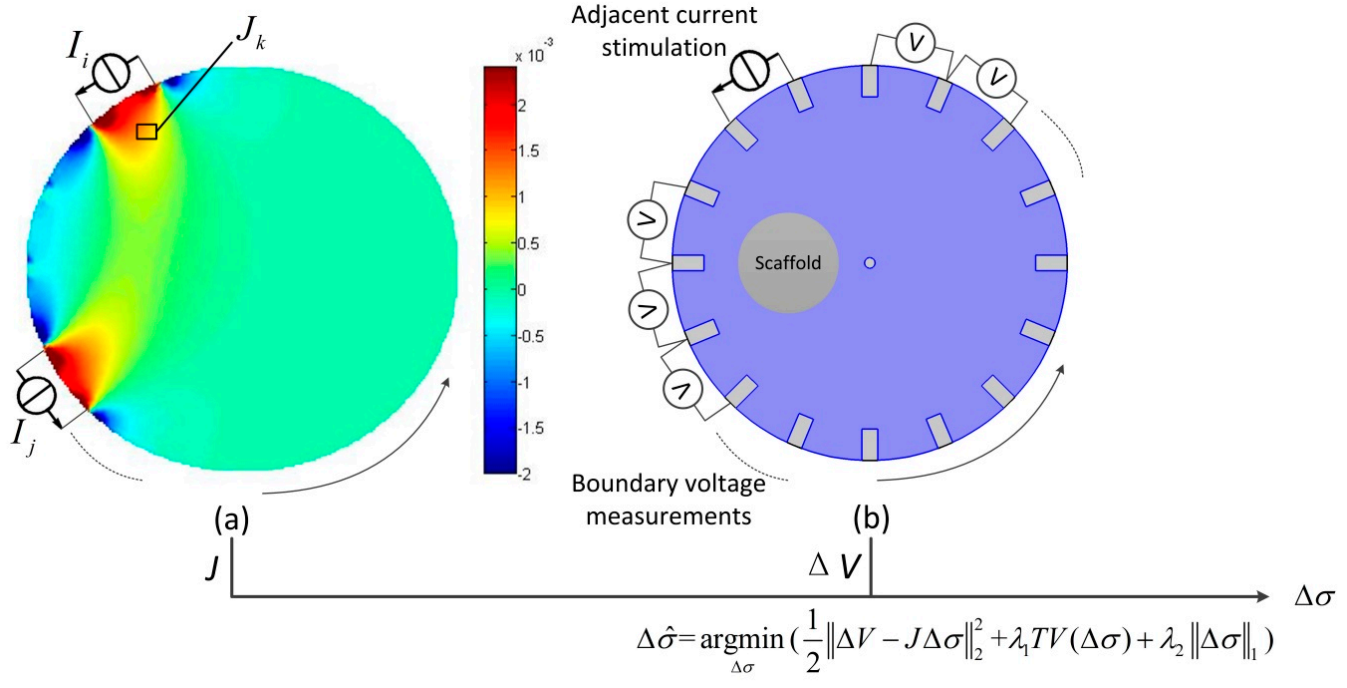
Schematic diagram showing the ways to reconstruct the conductivity distribution based on (**a**) the normalized sensitivity matrix $J_{i,j}$ for the measurement using electrode pairs $i^{th}$ and $j^{th}$, and (**b**) the boundary voltage variation ΔV. Each boundary measurement has a corresponding sensitivity matrix. The sensitivity $J_{k}$ indicates the amplitude of boundary voltage variation if the conductivity changes at the $k^{th}$ voxel. With the boundary voltage variation induced by the cells, the cell distribution in the scaffold can be reconstructed by minimizing the difference between the measurements and the estimated voltage variation, which is calculated by the sensitivity matrix J and the estimated conductivity variation $\Delta \hat{\sigma }$.

The forward problem corresponds to the prediction of the boundary voltage distribution when the conductivity distribution and the injected current are known. Solving the forward problem involves the calculation of the relationship between boundary voltages V (V) and the conductivity distribution σ (S/m), which can be represented by a derived formula from the Maxwell’s equation and be linearized in the finite element model [29]:

(1) ΔV=JΔσ

where J is the sensitivity matrix that indicates the relationship between the change in conductivity $\Delta \sigma_{k}$ (S/m) in the $k^{th}$ voxel and the change in the voltage distribution ΔV(V). It can be calculated in the simulation by stimulating a current in the sensor with a homogeneous solution and measuring the electric potential at different voxels (Figure 1a):

(2) $$J_{k}=\frac{dv_{i,j}}{d\sigma_{k}}=-\int_{voxel\;k}\nabla v_{k}(I_{i})\times \nabla v_{k}(I_{j})dxdy$$

where $\nabla v_{k}(I_{i})$(V/m) is the electric potential at the $k^{th}$ voxel when current I(A) is stimulated at the $i^{th}$ electrode pair, and $\nabla v_{k}(I_{j})$(V/m) is the electric potential at the $k^{th}$ voxel when the current I(A) is stimulated at the $j^{th}$ electrode pair.

The inverse problem corresponds to the calculation of the variation of the conductivity distribution based on the sensitivity matrix and the boundary voltage changes. The voltage changes (Figure 1b) can be measured with either the time-difference EIT method or the frequency-difference EIT method.

In the time-difference EIT, the current with a single frequency is injected into the sensor, and the measurement at $t_{0}$(s) is taken as a reference. Then, the reference is subtracted from the voltage distribution at $t_{1}$(s) to indicate the conductivity perturbation during this period:

(3) $$\Delta V=V_{t_{1}}-V_{t_{0}}$$

In the frequency-difference EIT, a pair of complementary currents with two frequency components, $f_{0}$(kHz) and $f_{1}$(kHz), are injected to the electrodes. The voltage difference between two frequencies is used for the image reconstruction to represent the frequency response of the tissue under test:

(4) $$\Delta V=V_{f_{1}}-V_{f_{0}}$$

The solution to the inverse problem is to solve the following least square function, which minimizes the difference between the measured voltages distributions and the estimated voltage distributions:

(5) $$\Delta \hat{\sigma }=\underset{\Delta \sigma }{\mathrm{argmin}}(\frac{1}{2}{\left\|\Delta V-J\Delta \sigma \right\|}_{2}^{2})$$

where $\Delta \hat{\sigma }$ (S/m) is the estimated conductivity variation. However, the inverse problem is “ill-posed”, and the solution to Equation (5) is not unique. To overcome the ill-posedness, the objective function, Equation (5), is constrained with an additional regularization function. In this study, the isotropic TV and l1 joint regularization [30] were applied:

(6) $$\Delta \hat{\sigma }=\underset{\Delta \sigma }{\mathrm{argmin}}(\frac{1}{2}{\left\|\Delta V-J\Delta \sigma \right\|}_{2}^{2}+\lambda_{1}TV(\Delta \sigma )+\lambda_{2}{\left\|\Delta \sigma \right\|}_{1})$$

where TV(Δσ) is the TV of the reconstructed image [31] and ${\left\|\Delta \sigma \right\|}_{1}$ is the l1 penalty term. $\lambda_{1}$ and $\lambda_{2}$ are the regularization parameters for the TV regularization and the l1 regularization, respectively.

### 2.2. EIT Measurements in the Miniature Sensor

**Figure 2:**
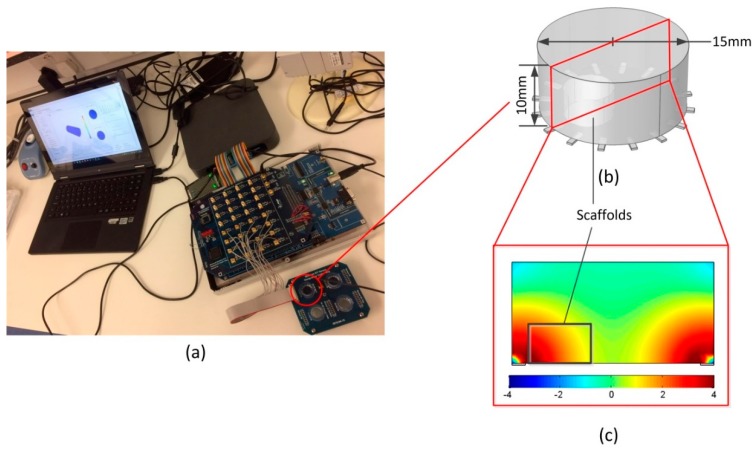
(**a**) An EIT system; (**b**) a sensor structure; and (**c**) the logarithmic full sensitivity matrix of the sensor at the vertical cross section.

The EIT measurements were carried out with a multi-frequency EIT system [32] and a miniature EIT sensor [33] developed at the University of Edinburgh (Figure 2a). The chamber of the sensor was a cylinder with a diameter and a height of 15 mm and 10 mm, respectively. Sixteen working electrodes were evenly distributed at the edge of the sensor (Figure 2b). In this study, we applied the adjacent method [13] for current stimulations and voltage measurements. A stimulation current of 1 mA with frequencies from 10 kHz to 100 kHz was first injected into the sensor through a pair of adjacent electrodes, and the corresponding voltages applied to other electrode pairs were successively measured with the system (Figure 1b). By switching the stimulation electrode pairs, this process generated a total of 104 independent measurements for the image reconstruction, which required approximately 0.07 s for a full scan. This measurement scheme provided high sensitivity to the conductivity variation at the bottom area, where the scaffold located (Figure 2c), so it was easier to detect the cell distribution. The selection of the current amplitude was based on the consideration of a heating effect [27], so that the cell viability was not affected by the injected current. The selection of the stimulation frequency was based on the three dispersion mechanisms introduced by Schwan [34], which illustrated the frequency response of biological tissues and cell suspensions. The α-dispersion appeared below several kHz and was not easy to measure due to the interference from electrode polarization effects. The β-dispersion distributed between 10 kHz to several MHz was mainly attributed to the interfacial polarization due to the existence of the insulating membrane surrounding the cells. The γ-dispersion was above 1 GHz and was related to the polarization of water molecules. The conductivity changes at 10–100 kHz were associated with cell membranes integrity (β-dispersion), and we could therefore estimate the cell distribution within the scaffolds based on the observed values [35]. 

### 2.3. Cell Culture

Breast cancer cells, MCF-7, were routinely cultured in a Dulbecco’s Modified Eagle’s Medium (Life Technologies, Carlsbad, CA, USA) containing 10% fetal bovine serum (Thermo Fisher Scientific, Waltham, MA, USA) and 1% penicillin-streptomycin (Life Technologies, Carlsbad, CA, USA). Cells were grown in an incubator at 37 °C with 5% CO_2_, and the medium was changed every 48 h. 

### 2.4. Cell Seeding

The cells were seeded in HyStem-HP hydrogels (Sigma-Aldrich, St. Louis, MO, USA) and 96-well AlgiMatrix scaffolds (Life Technologies, CA, USA) were used to accommodate the cells with a concentration of 5 × 10^6^ in each scaffold. This concentration allowed the cells to completely fill the void spaces of the scaffolds [36]. The equivalent volume fraction of the cells in the scaffold was 10.7%, which was lower than that of densely packed cell spheroids with a volume fraction between 46% and 65%, so we considered it as the sparse cell distribution. To prepare the hydrogel samples, HyStem-HP, Gelin-S and Extralink 2 powder were dissolved in the degassed water and mixed following the standard operation protocol to generate the HyStem-HP hydrogel solution. The cells cultured in monolayer, as mentioned in Section 2.3, were recovered with 0.25% trypsin (Mediatech, Manassas, VA, USA) and were centrifuged to cell pellets at 259 relative centrifugal force (RCF) for 5 min. The pellet was then suspended in the HyStem-HP hydrogel solution to achieve a cell concentration of 5 × 10^7^ per mL. One hundred microliters of cell suspension was added to the 96-well plate before gelation occurred. The blank hydrogel control was formed by adding 100 µL HyStem-HP hydrogel solution to the 96-well plate. To seed the AlgiMatrix scaffolds, the AlgiMatrix sponges were first rehydrated with 50% firming buffer for 5 min in the 96-well plate. The cell pellet was prepared with the same method mentioned above and was suspended with the culture medium to achieve a cell concentration of 5 × 10^7^ per mL. Each scaffold sample in the well was seeded with 100 µL cell suspension. The blank scaffold control was added to an empty well and was incubated with the samples. The 96-well plate was placed in the incubator to allow cell attachment. One hundred microliters of a medium was topped up to each well after 2 h. The samples and the blank controls were then transferred from the 96-well plate to the chamber of the miniature EIT sensor during the measurements after being cultured in the plate for 24 h.

## 3. Results

Time-difference and frequency-difference imaging for the hydrogels and the scaffolds were performed in this section. The sensor containing the cell-encapsulated hydrogel sample or scaffold sample was placed in the incubator during the measurements. Boundary voltages measurements were taken from the electrodes when multi-frequency stimulation current was injected into the sensor. In the control group, blank hydrogels and scaffolds were placed into the sensor and measured under the same conditions.

### 3.1. Time-Difference EIT Image Reconstruction

**Figure 3:**
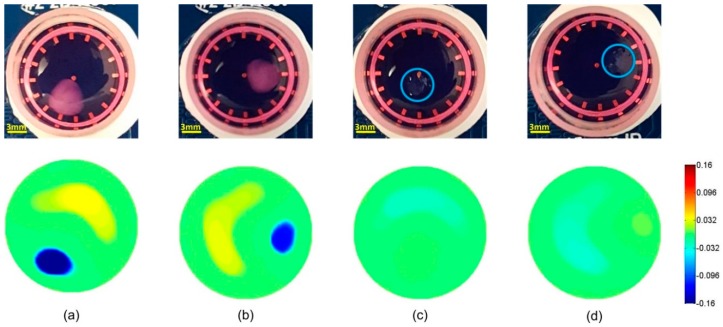
Time-difference EIT for a cell-loaded hydrogel sample at positions 1 (**a**) and 2 (**b**); and a blank hydrogel at similar positions 1 (**c**) and 2 (**d**). When no cells were present, blank hydrogels had a conductivity magnitude similar to that of the cell culture medium and were therefore below the noise level. In contrast, the presence of cells decreased the conductivity and resulted in a drop of 0.16 in conductivity. Non-specific change in conductivity was observed (yellow area) and was attributed to the change of liquid surface and liquid volume after the insertion of the hydrogel samples.

**Figure 4:**
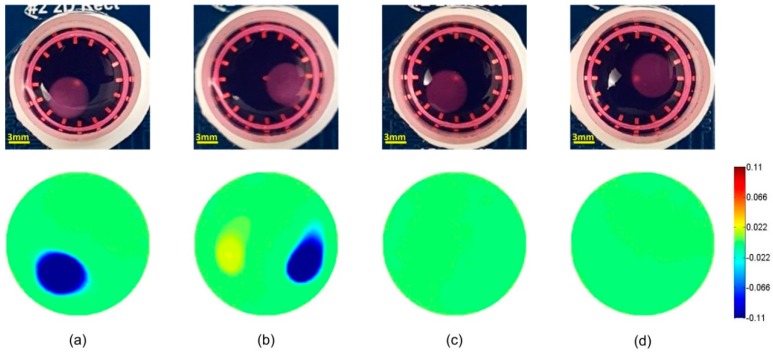
Time-difference EIT for a cell-loaded scaffolds sample at positions 1 (**a**) and 2 (**b**); and an independent blank scaffold at similar positions 1 (**c**) and 2 (**d**). When no cells were present, blank scaffolds had a conductivity magnitude similar to that of the cell culture medium and were therefore below the noise level. In contrast, the presence of cells decreased the conductivity and resulted in a drop of 0.11 in conductivity.

In the time-difference EIT, the boundary voltages for the samples and the homogeneous medium reference at 10 kHz were selected for image reconstruction. Figure 3 and Figure 4 show the time-difference images of the cells in hydrogels and scaffolds. The positions of the samples were correctly estimated based on the conductivity variation in the related area. The presence of cells in the hydrogels and the scaffolds resulted in a similar conductivity drop. Since the conductivity magnitude of the cells was lower than that of the medium [37], the presence of the cells reduced the local conductivity of the scaffolds, resulting in a drop in the image. The color bar indicated the relative change of local conductivity compared with the conductivity of the culture medium, which was a non-dimensional quantity. In Figure 3c,d and Figure 4c,d, the blank hydrogels and scaffolds could not be reconstructed in the images because the materials of hydrogels and scaffolds had a similar conductivity to that of the culture medium. The non-specific change in background conductivity was due to the introduction of hydrogels and scaffolds, which changed the liquid volume and the liquid surface in the sensor. As a consequence, the sensor models before and after scaffold insertion were different, so modelling error occurred.

### 3.2. Frequency-Difference EIT Image Reconstruction

In the frequency-difference EIT, the frequency range was set from 10 kHz to 100 kHz, and the voltage measurements at 10 kHz were used as $V_{f_{0}}$. We chose this frequency range to demonstrate the feasibility of EIT in the monitoring of the β-dispersions of the tissues [8], but alternative frequency ranges could be selected to show other distinct dispersion phases.

**Figure 5:**
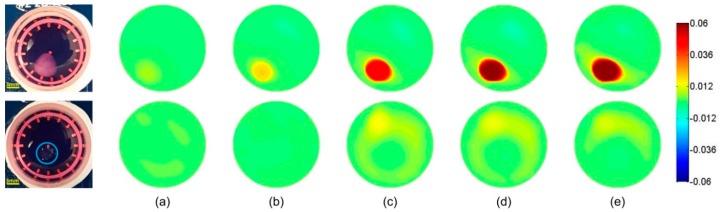
Differential frequency responses against 10 kHz for the hydrogel samples at position 1 at (**a**) 20 kHz, (**b**) 40 kHz, (**c**) 60 kHz, (**d**) 80 kHz and (**e**) 100 kHz. The first row is the cell-loaded hydrogel sample, and the second row is the blank hydrogel.

**Figure 6:**
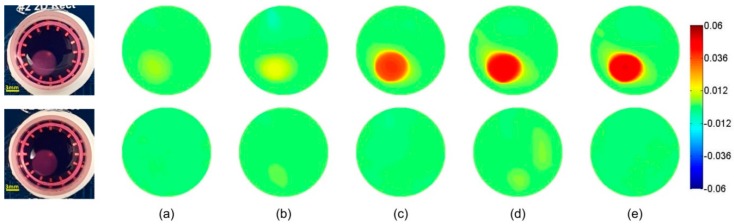
Differential frequency responses against 10 kHz for the AlgiMatrix scaffold samples at position 1 at (**a**) 20 kHz, (**b**) 40 kHz, (**c**) 60 kHz, (**d**) 80 kHz and (**e**) 100 kHz. The first row is the cell-loaded scaffold sample, and the second row is the blank scaffold.

Figure 5 and Figure 6 show the frequency-difference images of the hydrogels and the scaffolds. In Figure 5, the conductivity of the cell-loaded hydrogel sample increased from 0.007 at 20 kHz to 0.063 at 100 kHz. Since the conductivity variation of the hydrogels was similar to that of the culture medium, which was seen in the control groups, the conductivity increase was mainly attributed to the interfacial polarization across the cellular plasma membranes and their interactions with the extra- and intra-cellular electrolytes. In Figure 6, the conductivity of the cell-loaded scaffold sample increased from 0.006 at 20 kHz to 0.048 at 100 kHz, which was less than that of the hydrogels. When we referred to the Hanai’s equation [38], this variation meant that the volume fraction of cells in the hydrogel sample was higher than that in the scaffold sample. It was because that some cells were deposited on the bottom of the well through the pores of the scaffold before cell attachment, while all the cells in the suspension were encapsulated inside the hydrogel. Therefore, the actual cell concentration in the hydrogel sample was higher than that in the scaffold sample.

## 4. Discussion

The experiments in this study established the proof-of-principle that EIT could detect the presence of cells within 3D cell culture hydrogel scaffolds with an average thickness of 2.8 mm. Compared with the conventional techniques that require sectioning and dyeing as mentioned in the introduction, the EIT is a more convenient and more efficient method to monitor the cell distribution without destroying the samples. The time-difference EIT successfully reconstructed the local conductivity for both the hydrogels and the AlgiMatrix scaffold samples, allowing us to image the cell-loaded samples by mapping the local conductivity contrast due to the presence of MCF-7 cells. However, the background disturbances, such as the variation in the liquid volume, resulted in the non-specific conductivity error in the images.

For the purpose of this work, we reconstructed the frequency-difference EIT images based on the existence of the β-dispersion for biological tissues at radio frequencies. We postulated that, according to the Maxwell—Garnett theory, the presence of cells with a thin insulating membrane would result in frequency dependency of the sample conductivity. It could be detected by mapping the conductivity difference at frequencies across the frequency spectrum (10 kHz–100 kHz in our case). The results displayed in Figure 5 and Figure 6 are very promising and have the potential to provide more information, such as the cell concentration of the sample. 

The sensitivity of boundary measurements to the local conductivity contrast depended on the current density of the local area during the measurement. In Figure 2, it can be seen that the current density near the electrode was thousand times higher than that in the middle area. In order to increase the sensitivity, one effective method is to reduce the size of the sensor to fit the size of the scaffold. Reducing the size of the sensor increases the current density in the chamber with the same stimulation current, so the sensitivity in the whole space will be increased. Alternatively, increasing the number of independent measurements also allows a higher sensitivity to the changes of local conductivity. It could be achieved by using new sensor patterns such as the sensor with an increased number of electrodes [39,40]. Otherwise, rotational electrodes can be implemented to the sensor [41] so that the number of measurements can be increased while the size of the electrodes can be guaranteed, which minimizes the impact of contact impedance [42] to the voltage data.

At present, the spatial resolution of EIT is not as high as other imaging techniques, such as confocal and fluorescence microscopy. One of the most important factors is the low signal to noise ratio (SNR) in the measurement signal, which means that boundary voltage variation due to the fact that the conductivity variation of the tissue is small in comparison with the circuitry noise and the background interference. The application of frequency-difference imaging method penalizes the disturbance of liquid volume and liquid surface, reducing the non-specific errors in the reconstructed images. To further improve the spatial resolution, accurate system calibration [43] is required to compensate the circuitry noise before the experiment. Alternatively, advanced image reconstruction algorithms based on strong prior knowledge, such as the structure of the tissue, can be applied to improve the spatial resolution. 

For tissue engineering applications, it is highly desirable to monitor in real-time over long-term cell growth and differentiation, together with tissue formation in 3D scaffolds. When cell culture is performed over days, the conductivity of the culture medium is likely to change continuously over time, because of changes in dissolved gas content and pH change associated with the formation of cell by-products. In this case, the homogeneous reference is not available for time-difference EIT imaging. The frequency-difference EIT imaging proposed in this study overcomes this challenge, and it reconstructs images of the conductivity spectrum of tissues, which is only relevant to the frequency response of the cells. This method is more robust to the disturbance in the culture environment and is suitable to be applied in the long-term cellular assay.

## 5. Conclusions

In this paper, we confirmed that EIT is a suitable candidate to image and monitor viable cell distribution deep into 3D cell culture scaffolds. The EIT has the advantages of low cost, ease of operation, non-destructive and label-free features, and is suitable for long-term assays, but its performance will be better if its measurement error can be reduced in scaffold monitoring, because the sparse cell distribution produces a smaller conductivity variation of the area of interest, which results in a relative small SNR compared with conventional EIT applications. The application of frequency-difference imaging method mitigated the error caused by the change of liquid volume and liquid surface, so the non-specific background disturbance was eliminated. The application of the isotropic TV and l1 joint regularization algorithm maintained a clear boundary of the scaffold while further penalizing the background noise. Currently, we successfully reconstructed the cell distribution in the hydrogels and the scaffolds with an average thickness of 2.8 mm and the volume fraction of the cells at 10.7%. Future work should focus on the signal enhancement by optimizing the sensor design. Quantitative experiments with different seeding concentrations will be performed with the suggested optimizations to determine the limit of detection of EIT in the 3D sparse cellular assays, which is compared with that in the cellular metabolic assays.
